# ACPA Status Correlates with Differential Immune Profile in Patients with Rheumatoid Arthritis

**DOI:** 10.3390/cells10030647

**Published:** 2021-03-14

**Authors:** Achilleas Floudas, Mary Canavan, Trudy McGarry, Ronan Mullan, Sunil Nagpal, Douglas J. Veale, Ursula Fearon

**Affiliations:** 1 Molecular Rheumatology, Trinity Biomedical Sciences Institute, TCD, D02 R590 Dublin, Ireland; floudasa@tcd.ie (A.F.); mary.canavan3@gmail.com (M.C.); trudy.mcgarry@novartis.com (T.M.); 2 Department of Rheumatology, Tallaght University Hospital, D24 NR04 Dublin, Ireland; Ronan.Mullan@tuh.ie; 3 Janssen Research & Development, Immunology, Spring House, PA 19477, USA; sunil.x.nagpal@gsk.com; 4 EULAR Centre of Excellence, Centre for Arthritis and Rheumatic Diseases, St Vincent’s University Hospital, UCD, D04 N2E0 Dublin, Ireland; douglas.veale@ucd.ie

**Keywords:** RA, ACPA, B cells, T cells, synovial tissue

## Abstract

Rheumatoid arthritis (RA) is a progressive erosive autoimmune disease that affects 1% of the world population. Anti-citrullinated protein autoantibodies (ACPA) are routinely used for the diagnosis of RA, however 20–30% of patients are ACPA negative. ACPA status is a delineator of RA disease endotypes with similar clinical manifestation but potentially different pathophysiology. Profiling of key peripheral blood and synovial tissue immune populations including B cells, T follicular helper (Tfh) cells and CD4 T cell proinflammatory cytokine responses could elucidate the underlying immunological mechanisms involved and inform a treat to target approach for both ACPA-positive and ACPA-negative RA. Detailed high dimensionality flow cytometric analysis with supervised and unsupervised algorithm analysis revealed unique RA patient peripheral blood B cell and Tfh cell profiles. Synovial tissue single cell analysis of B cell subpopulation distribution was similar between ACPA− and ACPA+ RA patients, highlighting a key role for specific B cell subsets in both disease endotypes. Interestingly, synovial tissue single cell analysis of CD4 T cell proinflammatory cytokine production was markedly different between ACPA− and APCA+ RA patients. RNAseq analysis of RA patient synovial tissue highlighted disease endotype specific gene signatures. ACPA status associates with unique immune profile signatures that reinforce the need for a treat to target approach for both endotypes of RA.

## 1. Introduction

Rheumatoid arthritis is a chronic progressive autoimmune disease with a diagnosis that is based on several clinical and laboratory criteria, including the presence of auto antibodies, rheumatoid factor (RF) and ACPA. Serological evidence of ACPA can predate the onset of RA which highlights their potential involvement in disease pathogenesis [1]. Despite the high diagnostic value of ACPA, a proportion of 20–30% of RA patients are ACPA negative (APCA−) [2]. With 0.8% of healthy individuals harboring ACPA antibodies, other confounding factors in addition to ACPA facilitate the pathogenesis of RA, importantly, these could differ between ACPA positive (ACPA+) and ACPA− RA [3]. Genome-wide association studies have revealed genetic differences between ACPA− and ACPA+ RA, surprisingly these differences appear to be contained within the HLA encoding region [4]. Further evidence supports the hypothesis that ACPA status is indicative of two distinct endotypes of disease, radiographical differences consistent with ACPA+ RA being more erosive compared to ACPA− RA along with differences in the pattern of early joint involvement have been reported [5,6]. Differences in pre-clinical early RA symptoms between the two disease endotypes could be evidence of disparate development [7], with potential for different pathophysiology and specific treatment approaches. Indeed, results from the Combination Anti-Rheumatic Drugs in Early RA (CARDERA) trial, highlight a specific need for disease-modifying antirheumatic drugs (DMARDs) combined with corticosteroids for the prevention of radiographic progression of ACPA+ RA patients [8].

Elucidating the underlying immunological differences between the two disease endotypes could inform the design of specific treatment regimes. There is currently a paucity of data regarding potential immune profile differences associated with ACPA status in RA especially in relation to immune responses at the site of inflammation. It has recently been suggested that CCR6 memory CD4 T cells could play a role in ACPA+ RA due to their increase in peripheral blood of ACPA+ compared to ACPA− RA patients [9]. CCR6 is a marker of Th17 cells, in agreement with the aforementioned study, increased frequency of IFN-γ producing Th1 and IL-17A producing Th17 cells in the periphery of ACPA+ compared to ACPA− RA patients has been reported [10]. Immunohistology studies of synovial tissue have demonstrated conflicting results, with some studies showing an increase in synovial B cell lymphoid aggregates, and T cell subsets in ACPA+ RA associated with development of more erosive disease, while other studies showed no differences in immune cell infiltrates, but did demonstrate increased synovial cytokine responses [11,12,13,14]. Furthermore, studies have shown circulatory cytokine differences with an increase in levels of CXCL13 and CXCL12 in ACPA+ compared to ACPA− RA patients, with a recent study demonstrating an increase in CXCR3^+^ CD4 T cells in the synovial fluid compared to the periphery, irrespective of ACPA status [11,13,15].

In this study we have performed extensive characterization by high dimensionality flow cytometric analysis of several key immune populations in peripheral blood and importantly in single cell synovial tissue cell suspensions of ACPA+ and ACPA− RA patients, additionally, supervised and unsupervised algorithm analysis was used in order to identify disease specific immune profiles. We report peripheral blood B cell subpopulation distribution differences with a memory B cell bias for ACPA+ compared to ACPA− RA. Interestingly, a marked synovial accumulation of double negative (DN) and switched memory B cells was observed for both ACPA+ and ACPA− RA patients, indicative of the potentially key involvement of the aforementioned synovial B cell subpopulations in RA disease pathogenesis. Importantly, increased RA patient peripheral blood T follicular helper (Tfh) cell frequency and synovial tissue CD4 T cell pro-inflammatory cytokine profile and polyfunctionality were dependent on ACPA status.

The data presented herein strengthen the hypothesis that ACPA status is indicative of distinct disease endotypes in RA and highlight the potential for specific therapeutic approaches.

## 2. Materials and Methods

### 2.1. Synovial Tissue Single Cell Suspensions

RA patient arthroscopies were performed under local anaesthetic using Wolf 2.7 mm needle arthroscopy or ultrasound guided biopsy as previously described [16]. Synovial tissue isolation and generation of single cell suspension for further analysis was performed as described previously [17]. Briefly, approximately 15 synovial biopsies were isolated per patient and enzymatically and mechanically digested using the GentleMACS Tumor Dissociation Kit, human (Miltenyi Biotech, Bergisch Gladbach, Germany) as per manufacturers’ instructions. Briefly, biopsies are placed in RPMI supplemented with enzymes H, R and A in a gentleMACS™ C Tube followed by initial mechanical disruption of the tissue using program h_tumor_01 on a gentleMACS™ Dissociator. The samples are then incubated at 37 °C under continuous rotation using the MACSmix Tube Rotator for 1 h with further application of the gentleMACS™ Dissociator every 30 min. The single cell suspension is then generated by filtration of the digested synovial biopsies through a 70 µM cell strainer. Peripheral blood mononuclear cells (PBMC) and synovial fluid mononuclear cells (SFMC) were also isolated using a density gradient preparation as described previously [18]. Detailed clinical information data are provided in the Supplementary Tables S1–S3.

### 2.2. Flow Cytometric Analysis

RA patient PBMC, SFMC and synovial tissue single cell suspensions were used for flow cytometric analysis. Cells were washed in PBS followed by incubation with LIVE/DEAD fixable NIR (Thermofisher, Waltham, MA, USA) viability according to the manufacturer’s instructions. Cells were then washed with PBS and subjected to an Fc receptor block step (TruStain FcX Receptor blocking solution (Biolegend, San Diego, CA, USA)) and extracellular staining with antibody combinations for 30 min at 4 °C (Supplementary Table S4). Following incubation, cells were washed x2 in FACS buffer (PBS with 2% FBS and 0.002% *w*/*v* sodium azide, made in house, all reagents from Sigma). If intracellular staining was required, cells were subsequently fixed and permeabilised using the intracellular Foxp3 staining kit (eBiosciences, San Diego, CA, USA) as per the manufacturer’s instructions. Following the fixation and permeabilisation step, cells were washed in perm buffer and incubated with antibodies against intracellular targets for 30 min at 4 °C (Supplementary Table S4). Cells were then washed x1 with perm buffer and x1 with FACS buffer prior to acquisition on a 4 laser LSR Fortessa cytometer (BD). Analysis was performed on FlowJo (v10) and R using relevant packages and functions as described below. Key gating steps followed prior to specific population subgating included gating of cells based on forward and side scatter characteristics, two independent doublet cell exclusion steps, followed by gating of live cells. Florescent minus one (FMO) gating controls were used were appropriate.

### 2.3. RNAseq

RA patient stratification based on ACPA status was performed on previously obtained RNAseq analysis data of total RNA extracted from RA patient whole biopsy synovial tissue samples [19]. Briefly, quality of RNA was evaluated using an Agilent bioanalyzer followed by RNAseq by Q2 Solutions (Morrisville, NC, USA). Sequencing libraries were prepared on Truseq stranded total RNA using the Illumina Ribo-Zero protocol. Sequencing of pooled libraries was performed on an Illumina HiSeq 2000 and raw read quality was evaluated using FastQC. Raw reads were trimmed based on sequence quality and adaptors leading to an average number of clusters per sample of 8.9 × 10^7^. Reads were then aligned to the human reference genome b37.3 using STAR v2.4 [20]. Quantification of aligned reads was performed using RSEM v1.2.14 with the University of California Santa Cruz (UCSC) transcriptome model (accessed on 17 March 2014) that included lincRNAs from Ensembl v75. Aligned data were subjected to evaluation of quality utilizing several metrics including mapping rate, coverage and deviation from PCA.

### 2.4. Pathway Enrichment Analysis

Raw counts where analysed using the *DESeq2* (v1.28.1) pipeline in R for the identification of differentially expressed genes between ACPA+ and ACPA− RA samples. Pathway analysis was then performed using package *pathfindeR* (v1.6.1) in R with the most recently update KEGG database and a stricter to default adjusted *p* value enrichment threshold of 0.01, gene sets with 5 to 500 genes were considered and an enrichment threshold of 0.01 [21]. MA plots were generated using package *DESeq2* with *apeglm* shrinkage of the data in R. PCA analysis plots were generated in *R* on variance stabilized transformed data using function vst of package *DESeq2.* The effect of transformation on variance was assessed by generating SD to mean plots by using function *meanSdPlot(assay(ntd))* as part of package *DESeq2.*

### 2.5. Hierarchical Clustering and PCA

Hierarchical clustering and heatmap generation was performed on flow cytometric analysis data in *R v4.0* with function *scale* to generate scaled data and package *gplots v3.1.1*, function *heatmap.2*. PCA plots and biplots were generated under *R v4.0* with package *ggbiplot v0.5* with function *ggbiplot*.

### 2.6. Statistical Analysis

Statistical analysis was performed using Prism 7 (GraphPad) software, version 9.0. Unpaired two-tailed Mann–Whitney test and unpaired 2-tailed standard Student’s *t*-test were used as indicated. Statistical significance was considered with *p* values of less than 0.05.

### 2.7. Study Approval

Peripheral blood, synovial fluid and synovial tissue samples were collected from patients that were recruited from the Rheumatology Department, St. Vincent’s University Hospital, UCD and Tallaght University Hospital, TCD. Healthy control peripheral blood healthy volunteers recruited at Trinity Biomedical Sciences Institute and St. Vincent’s University Hospital. All subjects gave fully informed written consent approved by the institutional Ethics Committee and research was performed in accordance with the Declaration of Helsinki.

## 3. Results

### 3.1. Peripheral Blood B Cell Subpopulation Profile of ACPA− and ACPA+RA Patients

**Figure 1:**
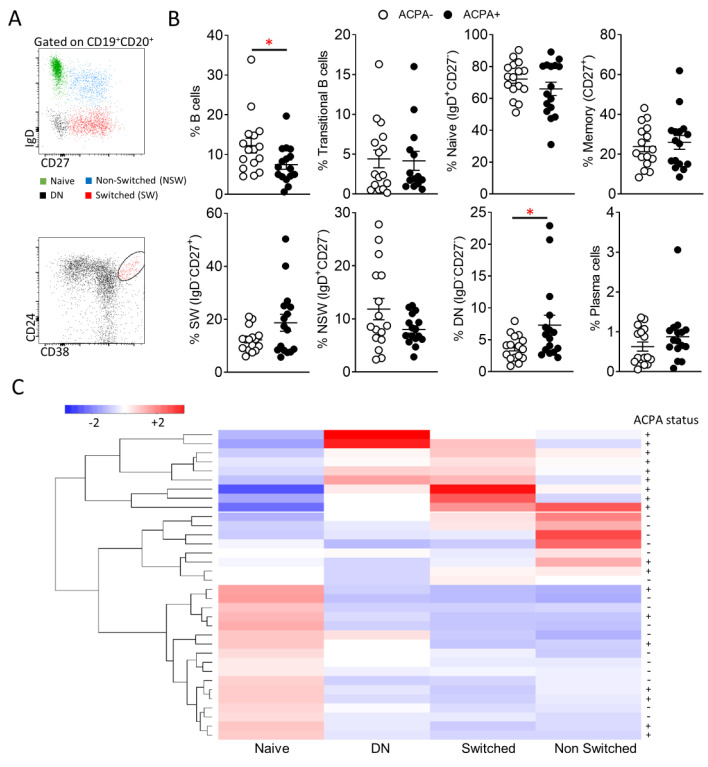
B cell subpopulation profile of ACPA− and ACPA+ RA patients. (**A**) Representative flow cytometric analysis for the identification of Naïve (IgD^+^CD27^−^), Non-switched memory (IgD^+^CD27^+^), Switched memory (IgD^−^CD27^+^), Double negative memory (IgD^−^CD27^−^) and transitional (CD24^++^CD38^++^) peripheral blood B cells. (**B**) Frequency of the indicated peripheral blood B cell subpopulations of ACPA (*n* = 16) and ACPA+ (*n* = 17) RA patients. (**C**) Hierarchical clustering of ACPA− and ACPA+ RA patients based on scaled frequency of 4 major B cell subpopulations. Data are presented as mean ± SEM, symbols represent individual samples. Statistical analysis was performed by using two-tailed Mann-Whitney test, *p* < 0.05 * were considered significant.

Key B cell subpopulations were identified by flow cytometric analysis of ACPA− and ACPA+ RA patient B cells. Following identification of CD19^+^CD20^+/−^ cells, the frequency of naïve (IgD^+^CD27^−^), non-switched (IgD^+^CD27^+^), switched (IgD^−^CD27^+^) and double negative memory (IgD^−^CD27^−^) B cells was assessed (Figure 1A,B). Total B cell frequency was significantly (* *p =* 0.04) reduced in ACPA+ compared to ACPA− RA patients (Figure 1B). ACPA+ RA patient peripheral blood B cell subpopulation distribution favoured memory B cell subpopulations with an increase in switched memory B cells and significantly increased DN memory B cells compared to ACPA− RA patients (* *p* = 0.04) (Figure 1B). Additionally, transitional B cell (CD24^++^CD38^++^) (enriched for IL-10 producing regulatory B cells) and plasma cell (CD138^+^CD27^++^) frequencies were examined (Figure 1B) [22]. While B cell subpopulation distribution between the two patient groups was comparable, in hierarchical clustering and PCA analysis of normalised flow cytometric data, trends were detectable with ACPA− B cell subpopulation distribution being skewed towards increased naïve B cells and ACPA+ towards increased switched and DN B cell frequency (Figure 1C and Figure S1).

### 3.2. Predominance of Switched and DN Memory B Cells in the Synovial Tissue of ACPA− and ACPA+ RA Patients

**Figure 2:**
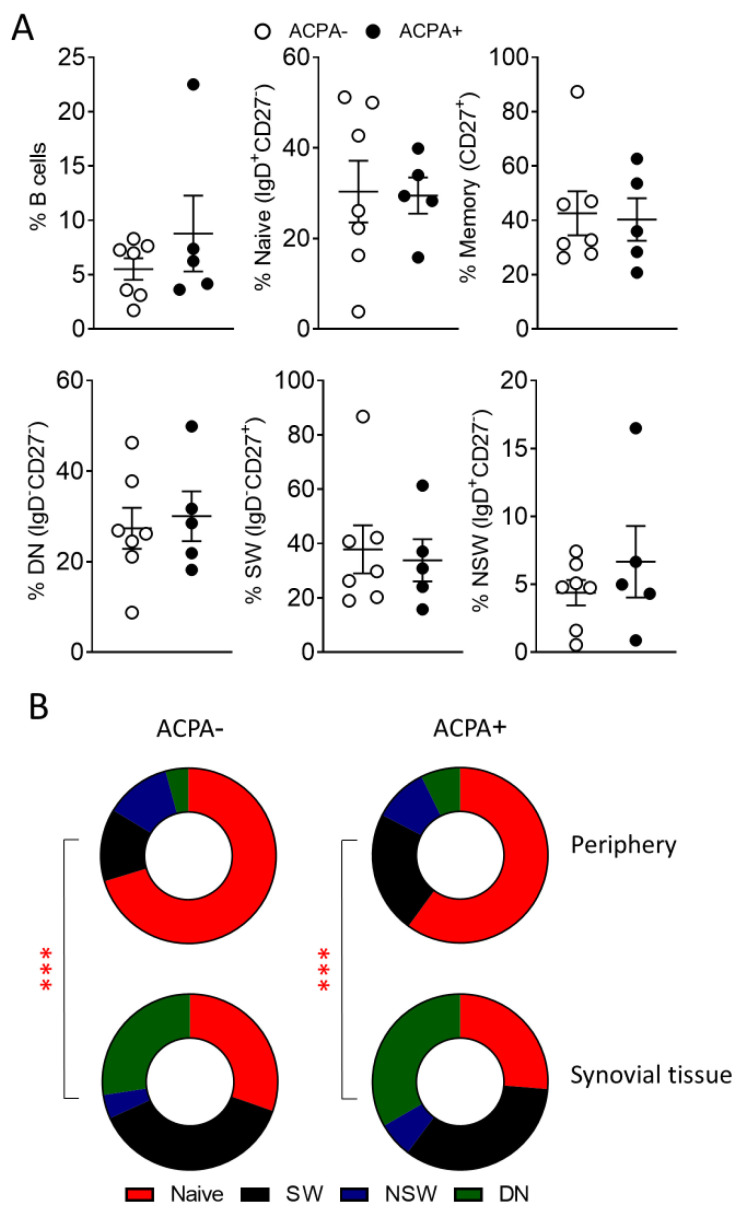
Synovial tissue B cells of ACPA− and ACPA+ RA patients. (**A**) Frequency of the indicated synovial tissue B cell subpopulations of ACPA− (*n* = 7) and ACPA+ (*n* = 5) RA patients. (**B**) Comparison of peripheral blood and synovial tissue B cell subpopulation frequency for ACPA− and ACPA+ RA patients, average p value range when comparing B cell subpopulations for the indicated groups is shown. Data are presented as mean ± SEM, symbols represent individual samples. Statistical analysis was performed by using two-tailed Mann-Whitney test, *p* < 0.001 ***.

ACPA− and ACPA+ RA patient synovial tissue single cell suspensions were subjected to flow cytometric analysis for the characterisation of B cell subpopulations. Surprisingly, B cell subpopulation distribution was similar between the two patient groups (Figure 2A), but differed significantly to peripheral blood B cells (Figure 2B). This is indicative of potentially key roles for switched and DN memory B cells in synovial inflammation irrespective of the ACPA status of the patient.

### 3.3. Increased Peripheral Blood T Follicular Helper (Tfh) Cell Frequency in ACPA+ Compared to ACPA− RA Patients

**Figure 3:**
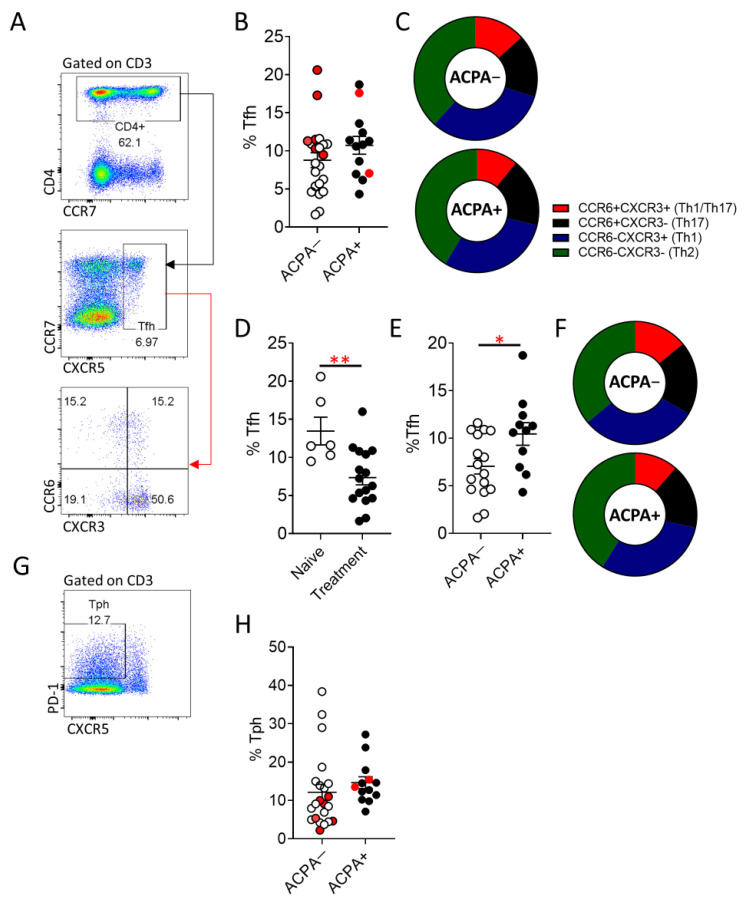
Increased Tfh cell frequency in ACPA^+^ compared to ACPA− RA patients undergoing treatment. (**A**) Representative flow cytometric analysis for the identification of Tfh cells (CXCR5^+^CD4^+^) and subsequent characterisation of CCR6^+^CXCR3^+^ (Th1/Th17-like Tfh), CCR6^+^CXCR3^−^ (Th17-like Tfh), CCR6^−^CXCR3^+^ (Th1-like Tfh) and CCR6^−^CXCR3^−^ (Th2 like Tfh), cells. (**B**) Cumulative data on the Tfh cell frequency of APCA− and APCA+ RA patients, samples in red indicate treatment naïve patients. (**C**) Tfh distribution to the indicated subpopulations. (**D**) Frequency of Tfh cells in treatment naïve compared to treated ACPA− RA patients. (**E**,**F**) comparison of total Tfh and subpopulation distribution between ACPA− and ACPA+ RA patients following exclusion of naïve to treatment patients. (**G**,**H**) Representative flow cytometric analysis for the identification of Tph cells (CD4^+^PD-1^+^CXCR5^−^) and peripheral blood Tph frequency of ACPA− and ACPA+ RA patients. Data are presented as mean ± SEM, symbols represent individual samples. Statistical analysis was performed by using two-tailed Mann-Whitney test, *p* < 0.05 * were considered significant, (*p* < 0.01 **).

Despite the observed similarities in synovial B cell subpopulation distribution between ACPA+ and ACPA− RA patients, autoantibody production could be driven by altered Tfh cell responses. Therefore, we examined peripheral blood Tfh (CXCR5^+^) cells and the recently described peripheral T helper cells (Tph) (CXCR5^−^PD-1^+^) (Figure 3) [23]. While Tfh cell frequency was comparable between ACPA+ and ACPA− RA (Figure 3A,B), naïve to treatment ACPA− RA patients had significantly (** *p* = 0.006) higher frequency of Tfh cells compared to patients undergoing treatment (Figure 3D). Interestingly, if naïve to treatment patients are excluded from the comparison between ACPA+ and ACPA− RA, ACPA+ patients maintain a significantly (* *p* = 0.03) higher Tfh cell population (Figure 3E). Expression of the chemokine receptors CCR6 and CXCR3 can inform on the cytokine bias of Tfh cells with CCR6^+^CXCR3^−^ Tfh sharing characteristics with Th17 cells, CCR6^−^CXCR3^−^ with Th2 cells, CCR6^−^CXCR3^+^ with Th1 cells and CCR6^+^CXCR3^+^ Tfh cells demonstrating a plastic Th1/Th17 phenotype (Figure 3A–F) [24,25]. The significantly higher frequency of Tfh cells in ACPA− compared to ACPA+ RA patients is not a result of an increase of a Tfh population with a specific cytokine bias nor is influenced by the treatment status of the patient (Figure 3C,E). Tph (CXCR5^−^PD1^+^) cells have recently been described as cells with an increased capacity to migrate towards sites of inflammation due to high expression of the chemokine receptors CCR2 and CCR5 and capable of promoting B cell antibody production through IL-21 [23]. No statistically significant difference, in the peripheral blood frequency of Tph cells in ACPA+ compared to ACPA− RA patients was identified (Figure 3G,H).

### 3.4. Synovial CD4 T Cell Proinflammatory Cytokine Differences Lead to Distinct Grouping of ACPA− and ACPA+ RA Patients

**Figure 4:**
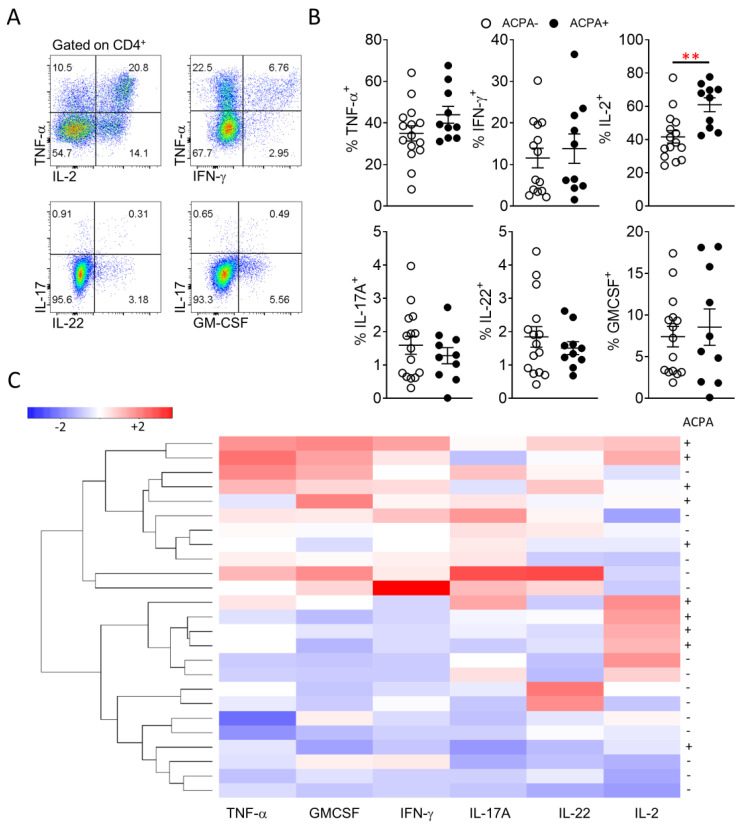
Similar peripheral blood CD4 T cell proinflammatory cytokine responses between ACPA− and ACPA+ RA patients. (**A**) Representative flow cytometric analysis for the identification of CD4 T cell proinflammatory cytokine responses. (**B**) Cumulative data for the CD4 T cell frequency of the indicated proinflammatory cytokines for ACPA− (*n* = 15) and ACPA+ (*n* = 10) RA patients. (**C**) Hierarchical clustering of ACPA− and ACPA+ RA patients based on scaled frequency of CD4 T cell cytokine production. Data are presented as mean ± SEM, symbols represent individual samples. Statistical analysis was performed by using two-tailed Mann-Whitney test, *p* < 0.05 were considered significant, (*p* < 0.01 **).

**Figure 5:**
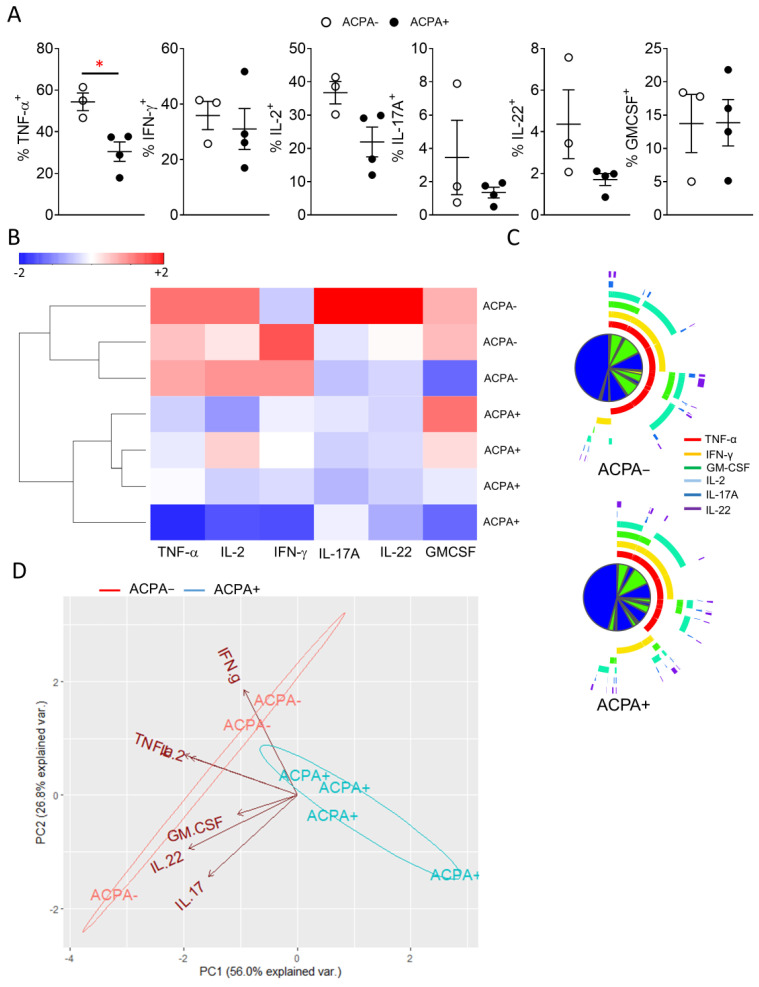
Distinct synovial tissue proinflammatory cytokine response profile for ACPA− compared to ACPA+ RA patients. (**A**) Cumulative data for the CD4^+^ T cell frequency of the indicated proinflammatory cytokines for ACPA− (*n* = 3) and ACPA+ (*n* = 4) RA patients (**B**) Hierarchical clustering of ACPA− and ACPA+ RA patients based on scaled frequency of CD4^+^ T cell cytokine production. (**C**) SPICE analysis of flow cytometric data for the identification of polyfunctional ACPA− and ACPA+ RA patient synovial tissue CD4^+^ T cells. (**D**) Biplot of PCA analysis on scaled flow cytometric data. Arrows indicate variable direction, ACPA− and ACPA+ RA patient synovial tissue CD4^+^ T cells cluster in 2 distinct groups as indicated by elliptical lines. Data are presented as mean ± SEM, symbols represent individual samples. Statistical analysis was performed by using two-tailed Students t test, *p* < 0.05 * were considered significant.

Several studies have previously indicated differential cytokine responses between ACPA+ and ACPA− RA patients, however results have been contradicting or lacking extensive direct description of proinflammatory T cell cytokine secretion [10,14,26]. While there is conflicting evidence regarding ACPA status and response to therapy, the comparative analysis of peripheral blood and importantly, synovial tissue T cell cytokine responses of ACPA− and ACPA+ RA patients, might allow the development of RA endotype specific treatment strategies [27]. Therefore, characterisation of T cell proinflammatory cytokine (TNF-α, IFN-γ, IL-2, IL-17A, IL-22, GM-CSF) expression was performed for peripheral blood and importantly synovial tissue CD4 T cells (Figure 4 and Figure 5). In peripheral blood, ACPA+ RA CD4 T cells showed significantly (** *p* = 0.003) higher expression of IL-2 and increased TNF-α compared to ACPA− RA patient CD4 T cells (Figure 4A,B). Unsupervised hierarchical clustering analysis of flow cytometric data, did not lead to a clear demarcation of ACPA− and ACPA+ RA peripheral blood CD4 T cell responses, indicative of a similar cytokine profile (Figure 4C).

Peripheral blood CD4 T cell proinflammatory cytokine responses are not however indicative of the local cytokine milieu at the site of inflammation and could therefore, provide reduced therapeutic potential compared to synovial tissue T cell responses. Following evaluation of synovial tissue CD4 T cell cytokine responses marked differences between ACPA+ and ACPA− RA were observed. Interestingly, ACPA− RA patient CD4 T cells show enhanced IL-2, IL-22 and significantly (* *p* = 0.01) increased TNF-α cytokine responses. Evaluation of the overall CD4 T cell cytokine milieu by unsupervised hierarchical clustering of flow cytometric data, led to a clear demarcation of ACPA− and ACPA+ RA patients with ACPA− demonstrating increased polyfunctionality and distinct grouping compared to ACPA+ RA patient synovial CD4 T cells (Figure 5B–D). While the synovial tissue CD4 T cell cytokine response comparison between ACPA− and ACPA+ RA is better served by the PCA analysis of Figure 5D, adding additional variables in the form of peripheral blood CD4 T cell responses and performing new PCA analysis shows the similarities of ACPA+ and ACPA− RA patient peripheral blood response and the separately grouped, more proinflammatory synovial responses influenced by increased expression of TNF-α, IFN-γ, IL-17A, IL-22 and GM-CSF but not IL-2 (Figure S2).

In order to assess if there are differences in Treg cells that could exacerbate the potential effect of the differential cytokine profiles observed between ACPA− and ACPA+ RA patients, peripheral blood and synovial fluid regulatory T cell frequency and memory status were examined (Figure S3). Peripheral blood Treg (CD127^−^ CD25^+^FOXP3^+^) frequency and naïve (CD45RO^−^CCR7^+^) to memory (CD45RO^+^CCR7^−^) distribution were similar between ACPA− and APCA+ RA patients (Figure S3A). In the synovial fluid Treg cell frequency is comparable, although there is a trend for reduced Treg frequency in APCA+ compared to ACPA− RA patients. Interestingly, the naïve to memory Treg cell distribution is significantly different with increased memory Treg cell frequency in synovial fluid of ACPA+ RA compared to ACPA− RA patients (Figure S3B).

### 3.5. Distinct Synovial Tissue Gene Signalling Pathways Are Implicated in ACPA+ Compared to ACPA− RA

T cell proinflammatory cytokine production while distinct between ACPA− and ACPA+ RA are one aspect of the underlying immume mechamisms that govern synovial inflammation. In order to futher deliniate the differnces in synovial tissue pathogenesis of ACPA− and ACPA+ RA we performed differential gene expression and pathway enrichment analysis of RNAseq data from 14 ACPA− and 32 ACPA+ RA patient synovial biopsies from study GSE89408 using the *DESeq2* pipeline in R [19].

**Figure 6:**
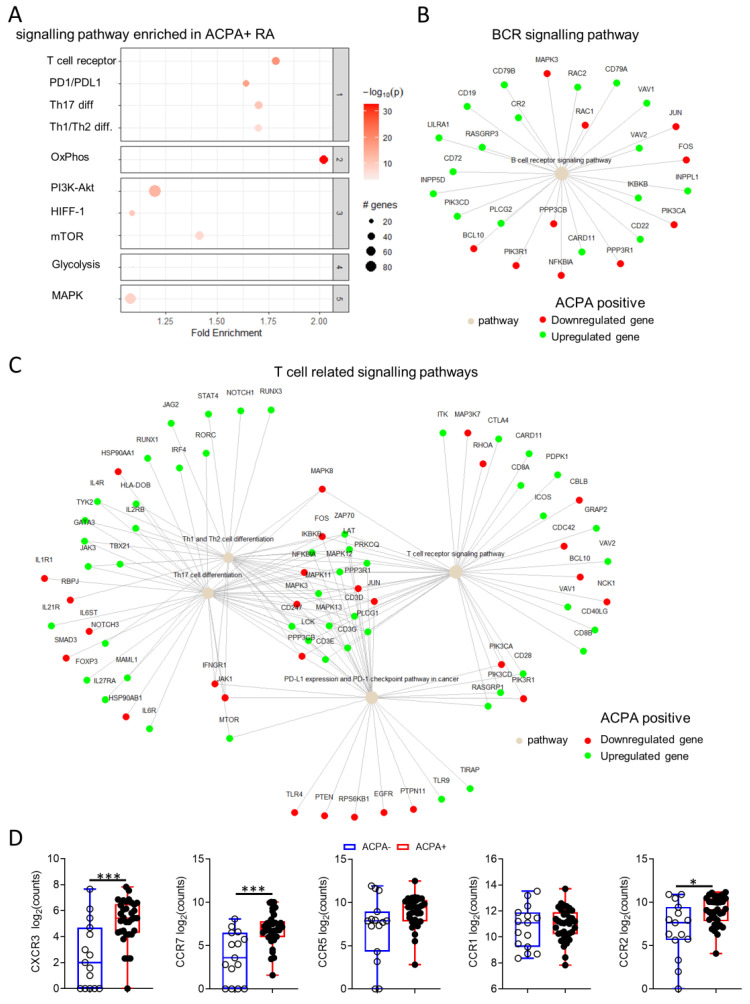
Distinct pathway enrichment in ACPA− and ACPA+ RA patient synovial tissue. (**A**) Enrichment plot of selected pathways enriched in APCA+ compares to ACPA- RA patient synovial tissue. (**B**) B cell receptor signalling pathway term plot showing specific gene up- or downregulation in ACPA+ compared to ACPA− RA patient synovial tissue. (**C**) Term plot of specific gene up- or downregulation for the top 3 most upregulated T cell dependent pathways in ACPA+ compared to ACPA− RA patient synovial tissue. (**D**) Data (log2(counts+1)) are presented as Box and whiskers plots (min to max), symbols represent individual samples. Statistical analysis was performed by using two-tailed Mann-Whitney test, *p* < 0.05 * were considered significant (*p* < 0.01 ***).

Of 19484 genes detected, 6198 (32%) were differentially expressed between ACPA− and APCA+ RA patient synovial tissue, with 4967 of them having known interactions (Figure 6 and Figure S4A). Pathway enrichment analysis was performed based on differentially expressed genes with an adjusted *p* value below 0.01 using a protein-protein interaction subnetwork analysis approach (*pathfindeR* in R) and the latest KEGG database [28,29]. Pathway enrichment returned a total of 194 pathways with significant differences in fold enrichment scores. Selected pathways with previously known involvement in RA pathogenesis are shown (Figure 6). Interestingly, increased enrichment of the OxPhos (Fold enrichment = 2.02) pathway and only a minor enrichment of the Glycolysis/Gluconeogenesis (Fold enrichment = 1.1) pathway was observed in ACPA+ compared to ACPA− RA (Figure 6A). The B cell receptor pathway and key T cell related pathways including the TCR signaling pathway were significantly enriched, a breakdown of the up- or downregulation of the genes involved in these pathways are shown (Figure 6B,C). Interestingly, differences were observed in the usage of chemokine receptors with significantly increased expression of CXCR3, CCR7 and CCR2 (* *p* = 0.004, ** *p* < 0.003 and * *p* = 0.04, respectively) in ACPA+ compared to ACPA− RA, highlighting potentially fundamental differences in immune cell recruitment between for the two endotypes of disease (Figure 6D).

Due to a small degree of observed heteroskedasticity of the data at low mean counts and in order to conform with the homoskedastic data assumption for downstream clustering and PCA analysis, data transformation with Variance Stabilizing Transformation (VST) was performed in R (package DESeq2, function vst) (Figure S4B) [30]. While there is a small degree of separation of the data based on PC1, there is no clear global grouping of ACPA+ and ACPA− RA patient synovial tissue RNAseq data (Figure S5).

## 4. Discussion

Genomic and radiographic differences and importantly differential response to treatment highlight the need for a treat to target approach of ACPA− and ACPA+ RA patients. Herein we have presented evidence of potentially distinct underlying immunological mechanisms involved, as well as points of convergence in the pathogenesis of the two disease endotypes of RA. While peripheral blood B cell subpopulation characteristics show a degree of separation based on the patients ACPA status, surprisingly the synovial tissue B cells are dominated by switched and double negative memory B cells irrespective of the ACPA status of the patient. Autoantibody production might be driven, however, by Tfh cell responses. Indeed, in ACPA+ RA patients there is significantly higher frequency of Tfh cells compared to ACPA−. ACPA specific B cells produce non class-switched antibody isotypes with increased somatic hypermutation with a gene signature that is associated with T cell-dependent responses [31]. Previous studies have suggested that increased Tfh cell responses in RA are driving B cell autoantibody production, therefore, the increased availability of Tfh cells in ACPA+ over ACPA− RA patients could be implicated in the generation of ACPA, however, further studies are required in order to elucidate the Tfh-autoreactive B cell interactions in RA [32,33].

Previous studies have shown conflicting results regarding the immune cell infiltrate in ACPA− and APCA+ RA synovial tissue based on immunohistological analysis [11,13,14]. This could be a result of markers used for the identification of infiltrating cells, cohort size or assay of choice, however, the overall consensus has hinted at chemokine and proinflammatory cytokine differences between ACPA− and APCA+ RA, but there has been limited investigation of the potential for differential proinflammatory cytokine T cell responses and polyfunctionality [11,13,14]. In this study, we have identified an increased CD4 T cell proinflammatory cytokine profile with a significant increase in TNF-α expressing cells and marked polyfunctionality in the synovial tissue of ACPA− compared to ACPA+ RA patients. CD4 T cell polyfunctionality is a strong correlator with disease progression and synovial inflammation [18,34]. While, ACPA have been identified in healthy individuals (0.8% of the population) and have been described in patients up to 14 years prior to the onset of RA, the diagnostic relevance of the observed T cell polyfunctionality differences between ACPA− and ACPA+ RA are limited [3,35]. Importantly, however, the differential cytokine profile, clearly separates ACPA− from ACPA+ RA and could have implications in the implementation of cytokine targeting treatments in RA. In addition to T cell polyfunctionality, the synovial fluid Treg cell compartment was divergent with increased memory Treg cell frequency in ACPA+ compared to ACPA− RA. The aforementioned observation warrants further investigation since memory Treg have previously been reported to have unstable FOXP3 expression with altered suppression and migratory characteristics compared to naive Treg cells [36,37]. Potential differences in disease pathogenesis between APCA− and ACPA+ RA are further illustrated by the specific pathway enrichment in ACPA+ compared to ACPA− RA patient synovial tissue. The BCR signaling and several key T cell related pathways including the TCR signaling and PD-1 pathway are preferentially enriched in ACPA+ compared to ACPA− RA. Despite the discrepancy between proinflammatory cytokines and enrichment of T cell specific pathways in ACPA+ compared to ACPA− RA, GM-CSF, IFN-γ and TNF-α showed, log2 fold changes indicating increased expression in ACPA− compared to ACPA+ RA synovial tissue biopsies highlighting the need for further characterization of T cell responses in RA depending on ACPA status.

Potential metabolic differences can serve as indicators of fundamental changes in disease pathogenesis, as enrichment of the OxPhos compared to the glycolysis pathway is evident in the ACPA+ to ACPA− RA comparison and should be investigated further. Differences in synovial tissue inflammation in ACPA+ and ACPA− RA could be present at the very early stages of pathogenesis as a result of differential cell recruitment due to distinct chemokine utilization. Previous studies have shown differences in chemokine utilization with increased IL-8, CXCL13 and CXCL12 [13,38]. This study reports ACPA+ RA synovial tissue specific increase in CXCR3, CCR7 and CCR2. While CCR2 blockade has failed in RA clinical trials, previous studies support CCR7 as a therapeutic target in RA, thus the increased expression of CCR7 in ACPA+ compared to ACPA− RA synovial tissue might result in increased sensitivity of ACPA+ RA to CCR7 inhibition [39,40]. Blockade of CXCR3 has been effective in suppressing arthritis progression in murine studies [41].

While the data presented herein, show differences between ACPA− and ACPA+ RA patients, further studies based on patient’s APCA titer may provide more evidence. Such studies would, however, require significantly larger cohorts of patients which in turn makes performing RNAseq analysis a considerable challenge. Treatment and disease severity were not significantly different between our 2 patient groups, however in other studies these factors may have been confounding. Further studies should be performed to elucidate the effect of treatment, particularly long term treatment with emphasis on response vs. no response, on the underlying immunological mechanisms of ACPA− and APCA+ RA. While it would be preferable to perform flow cytometric and RNAseq analysis on the same synovial tissue samples, it must be appreciated that both these techniques require significant numbers of tissue/cells which makes this approach technically very challenging. The characteristics of the synovial tissue samples used, however for RNAseq and flow cytometric analysis, were comparable for both patient groups.

The data presented herein, are indicative of key differences in the underlying immune mechanisms and disease pathogenesis of ACPA+ and ACPA− RA and raise the potential for a treat to target approach based on stratification of the patients on ACPA status.

## Data Availability

RNAseq data have been deposited at the European Nucleotide Archive as previously described (ENA-SRP092408) [19].

## Supplementary Materials

The following are available online at https://www.mdpi.com/2073-4409/10/3/647/s1, Figure S1: Grouping of ACPA− and ACPA+ RA patients based on B cell subpopulation distribution. Figure S2: Grouping of ACPA− and ACPA+ RA patient peripheral blood and synovial tissue CD4^+^ T cells based on pro-inflammatory cytokine production. Figure S3: Peripheral blood and synovial fluid regulatory T cell frequency of ACPA− and ACPA+ RA patients. Figure S4: RNAseq data transformation and variance shrinkage for downstream analysis and clustering. Figure S5: Similarity matrix and PCA analysis of synovial tissue RNAseq data. Table S1: Clinical information of RA patients for synovial tissue samples included in the flow cytometric analysis of Figure 2 and Figure 5. Table S2: Clinical information of RA patients for peripheral blood samples included in the flow cytometric analysis of Figure 1, Figure 3 and Figure 5. Table S3: Clinical information of RA patients for synovial tissue samples included in the RNAseq analysis of Figure 6. Table S4: Characteristics of antibodies used for flow cytometric analysis.
