# Supplementary material for: ACPA Status Correlates with Differential Immune Profile in Patients with Rheumatoid Arthritis
Source: Cells. 2021 Mar 14;10(3):647. doi: 10.3390/cells10030647 (PMC8000255; doi:10.3390/cells10030647)
Supplement: Supplementary file 1 [file cells-10-00647-s001.pdf]

Table 1. Clinical information of RA patients for synovial tissue samples included in the flow cytometric analysis of Figures 2 and 5. C-reactive protein (CRP), erythrocyte sedimentation rate (ESR), swollen 28-joint count (SJC28), tender 28-joint count (TJC28) and visual analogue scale (VAS) and disease activity score-28 (DAS28) at time of arthroscopy are shown.

Figure	ACPA	CRP	ESR	SJC28	TJC28	VAS	DAS28	Treatment
**2**	-	19.7	30	2	0	40	3.01	DMARD (Mtx)
**2**	-	<1	-	2	2	100	3.57	Tocilizumab
**2**	-	53.6	98	4	6	60	1.39	DMARD (Mtx)
**2**	-	-	-	1	0	10	5.18	Naïve
**2**	-	24	30	1	1	50	3.67	Naïve
**2**	-	1	2	0	5	40	3.02	DMARD (Mtx)
**2**	-	42	31	0	10	80	5.2	Naïve
**2**	+	8	43	0	3	80	3.84	DMARD (Mtx)
**2**	+	10.4	3	0	12	50	4.48	DMARD (Mtx)
**2**	+	6	17	0	1	12	2.39	DMARD (Mtx)
**2**	+	17	11	3	4	70	4.59	DMARD (Mtx)
**2**	+	3	12	2	2	40	3.57	DMARD (Mtx)
**5**	-	19.7	30	2	0	40	3.01	DMARD (Mtx)
**5**	-	53	98	2	2	100	3.57	DMARD (Mtx)
**5**	-	3	18	4	6	60	5.18	Rituximab
**5**	+	18	16	0	14	50	4.82	DMARD (Mtx)
**5**	+	3	12	2	2	40	3.57	DMARD (Mtx)
**5**	+	33.2	40	8	16	80	6.38	Rituximab
**5**	+	7.7	19	4	4	70	4.4	DMARD (Mtx)

Table S2: Clinical information of RA patients for peripheral blood samples included in the flow cytometric analysis of Figures 1, 3 and 5. Treatment groups (absolute number and %) and DAS28 (average per group and standard deviation are shown).

Figure	ACPA	Naive	DMARD	Biologics	DAS28
**1**	-	5 (31.25%)	10 (62.5%)	1 (6.25%)	4.1 (1.52)
**1**	+	1 (6.25%)	12 (75%)	3 (18.8%)	4.2 (2.15)
**3**	-	6 (27.27%)	15 (68.2%)	1 (4.5%)	3.9 (1.3)
**3**	+	2 (15.4%)	10 (76.9%)	1 (7.7%)	4.2 (2.34)
**4**	-	7 (46.6%)	7 (46.6%)	1 (6.6%)	3.9 (1.3%)
**4**	+	2 (20%)	6 (60%)	2 (20 %)	5 (1.7%)

Table S3: Clinical information of RA patients for synovial tissue samples included in the RNAseq analysis of Figure 6. Treatment groups (absolute number and %) and DAS28 (average per group and standard deviation are shown).

RNAseq	Naive	DMARD	DAS28
**ACPA negative**	6 (43%)	8 (57%)	4 (1.2)
**ACPA positive**	24 (75%)	8 (25%)	4.7 (1.03)

Table S4: Characteristics of antibodies used for flow cytometric analysis. Sample acquisition was performed on a 4-laser BD LSR Fortessa II cell analyser.

**Target**	**Conjugate**	**Clone**	**Supplier**
CD38	Alexa Fluor 488	HIT2	BioLegend
CD24	PerCP-Cy5.5	ML5	BD Biosciences
CD20	APC	2H7	BD Biosciences
CD27	Brilliant Violet 421	O323	BioLegend
IgM	Brilliant Violet 510	G20-127	BD Biosciences
CD138	Brilliant Violet 605	MI15	BioLegend
CD45	Brilliant Violet 650	H130	BioLegend
CD19	Brilliant Violet 711	HIB19	BioLegend
CD40	PE-CF594	5C3	BioLegend
IgD	PE-Cy7	1A6-2	BioLegend
PD-1	BV421	NAT105	BioLegend
PD-1	PE	NAT105	BioLegend
CD45	PE-Cy5	H130	BioLegend
CD45	BV510	H130	BioLegend
CXCR3	PE	G025H7	BioLegend
CXCR3	BV650	G025H7	BioLegend
CD3	BV786	HIT3a	BioLegend
CCR7	PE-CF594	G043H7	BioLegend
CD8a	PE-Cy5	HIT8a	BioLegend
CD4	PE-Cy7	RPA-T4	BioLegend
CCR6	BV711	G034E3	BioLegend
CXCR5	BV785	J252D4	BioLegend
IL-17A	BV650	BL168	BD Biosciences
GM-CSF	PE-CF594	21C11	BioLegend
TNFα	PercP-Cy5.5	Mab11	eBioscience
IFNγ	APC	B27	BD Biosciences
FOXP3	PE	206D	BioLegend

IL-2	BV605	MQ17H12	BioLegend
CD161	BV785	HP-3G10	BioLegend
CD161	PE-Cy7	HP-3G10	eBioscience
CD25	BV711	BC96	BioLegend
CD127	BV650	A019D5	BioLegend
CD39	PercP-Cy5.5	A1	BioLegend

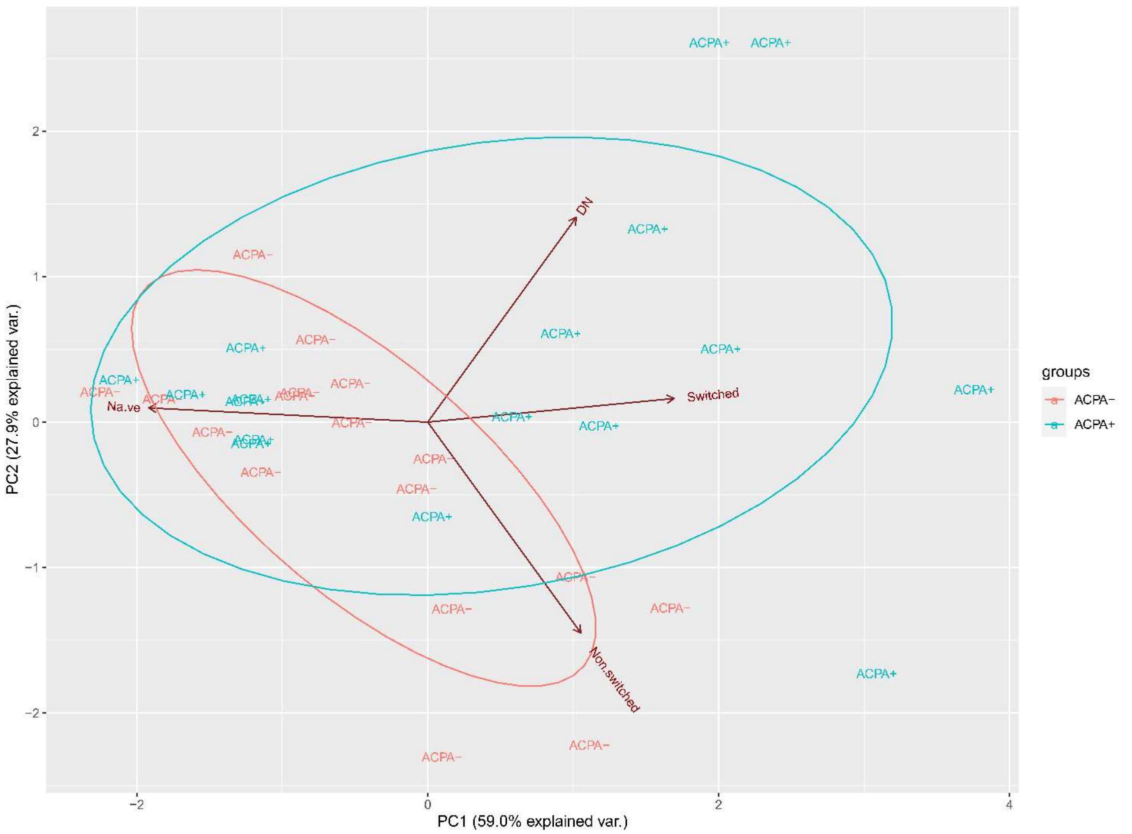

Figure S1. Grouping of ACPA− and ACPA+ RA patients based on B cell subpopulation distribution.

Biplot of PCA analysis on scaled flow cytometric data. Arrows indicate variable direction.

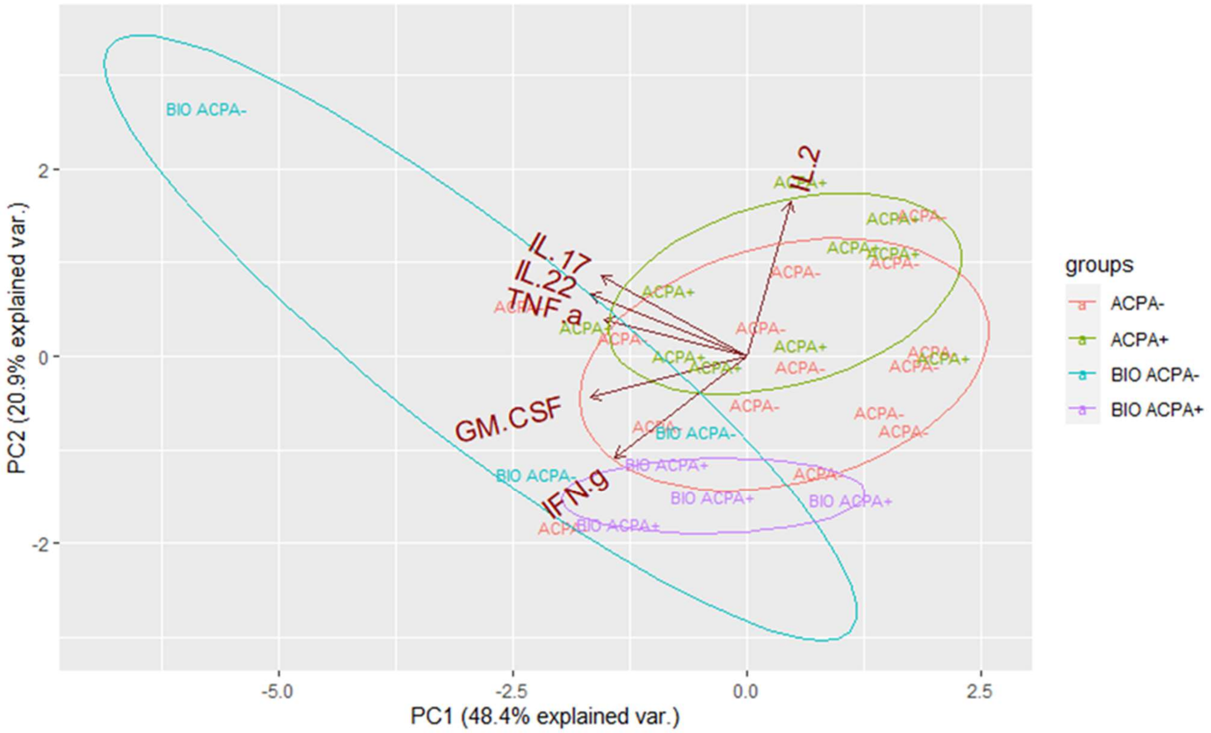

Figure S2. Grouping of ACPA− and ACPA+ RA patient peripheral blood and synovial tissue CD4⁺ T cells based on pro-inflammatory cytokine production.

Biplot of PCA analysis on scaled flow cytometric data. Arrows indicate variable direction.

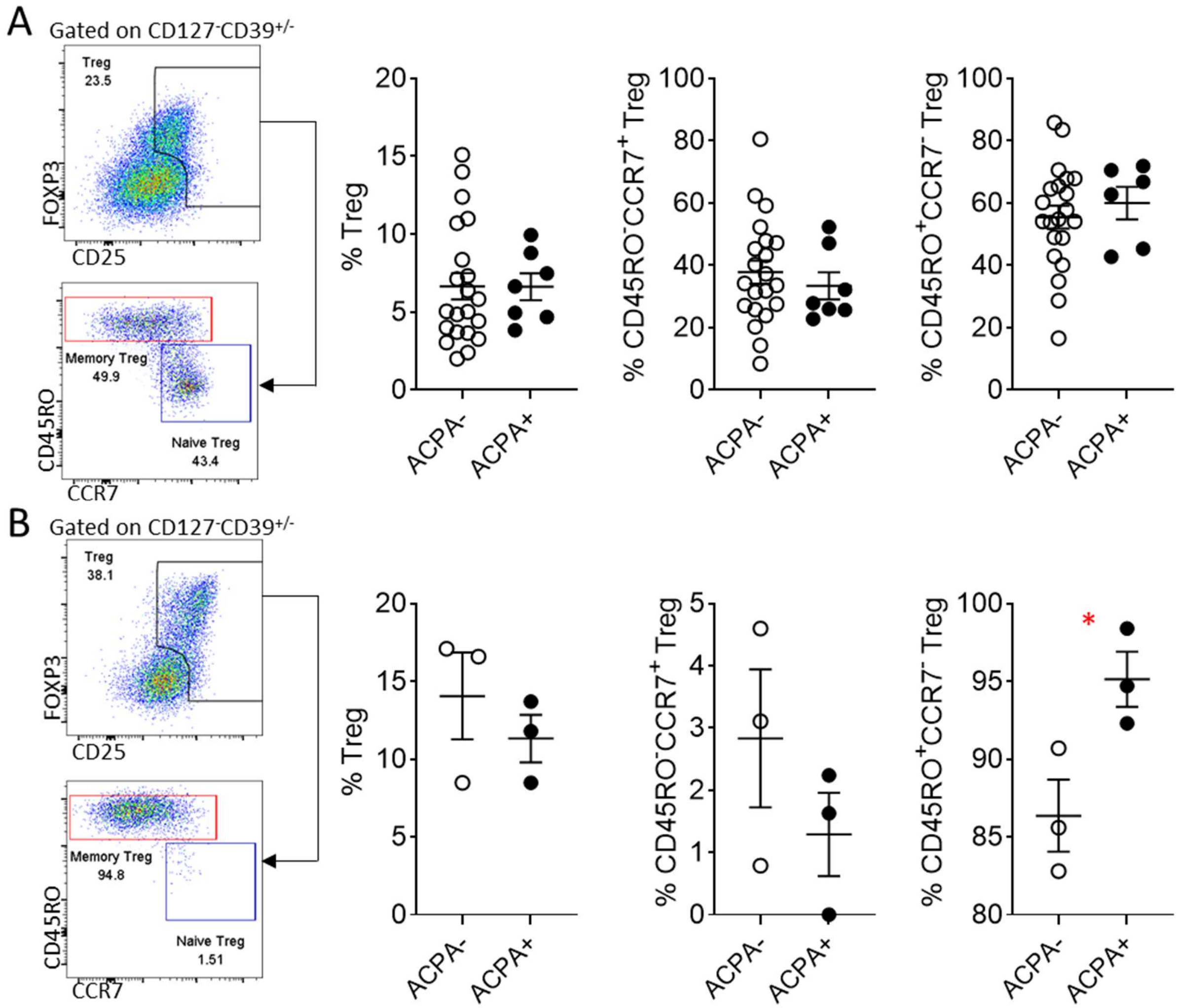

Figure S3. Peripheral blood and synovial fluid regulatory T cell frequency of ACPA- and ACPA+ RA patients.

A. Representative flow cytometric analysis for the identification and characterisation of peripheral blood Tregs and cumulative data on Treg frequency for ACPA- (n=21) and ACPA+ (n=7) RA patients. B. Representative flow cytometric analysis for the identification and characterisation of synovial fluid Tregs and cumulative data on Treg frequency for ACPA- (n=3) and ACPA+ (n=3) RA patients. Data are presented as mean ± SEM, symbols represent individual samples. Statistical analysis was performed by using two-tailed Mann-Whitney test, $p<0.05$* were considered significant.

A

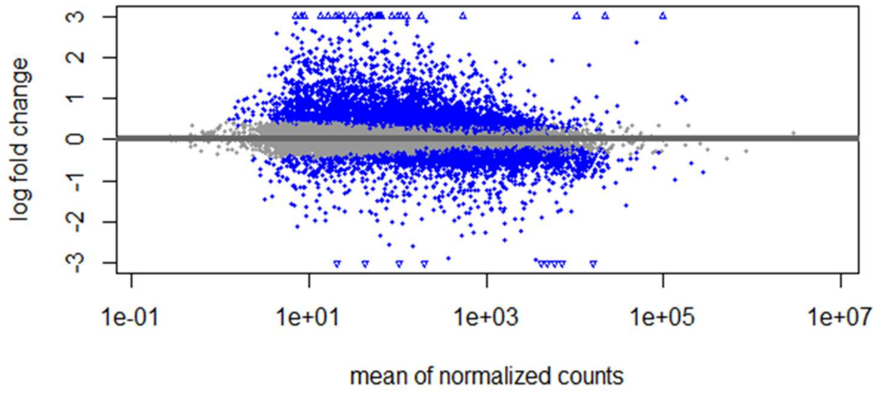

B

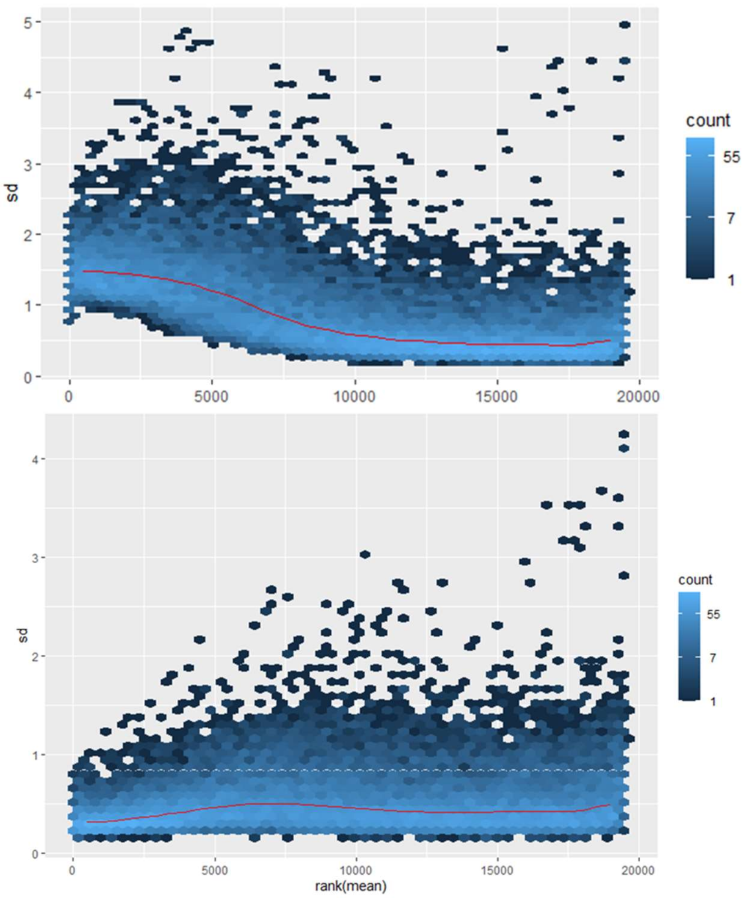

Figure S4. RNAseq data transformation and variance shrinkage for downstream analysis and clustering.

A. MA plot following apeglm data shrinkage in R, data package DESeq2. B. Variance (SD) and mean without (top) or with Variance Stabilizing Transformation (VST). VST corrects for increased variance linked to low mean values

A

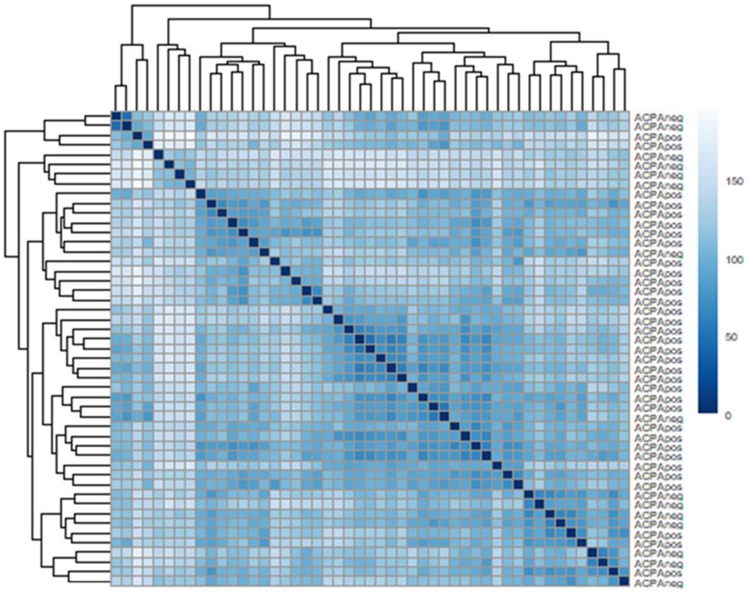

B

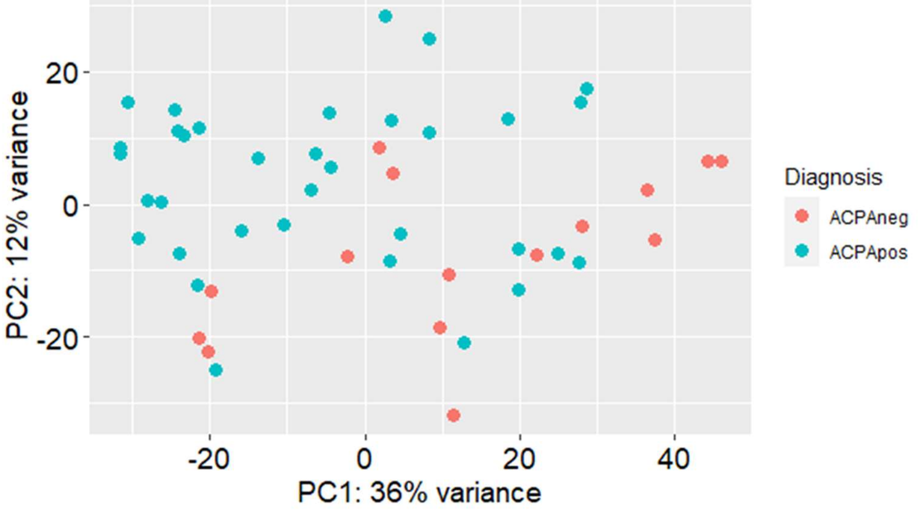

Figure S5. Similarity matrix and PCA analysis of synovial tissue RNAseq data.

A. Similarity matrix and B PCA plot of ACPA- and ACPA+ RA patient synovial tissue RNAseq data following vst transformation.
